# Quality of Prenatal and Childhood Diet Predicts Neurodevelopmental Outcomes among Children in Mexico City

**DOI:** 10.3390/nu10081093

**Published:** 2018-08-15

**Authors:** Ashley J. Malin, Stefanie A. Busgang, Alejandra J. Cantoral, Katherine Svensson, Manuela A. Orjuela, Ivan Pantic, Lourdes Schnaas, Emily Oken, Andrea A. Baccarelli, Martha M. Téllez-Rojo, Robert O. Wright, Chris Gennings

**Affiliations:** 1 Department of Environmental Medicine and Public Health, Icahn School of Medicine at Mount Sinai, 17 East 102nd Street, New York, NY 10029, USA; Stefanie.Busgang@mssm.edu (S.A.B.); Katherine.Svensson@mssm.edu (K.S.); Robert.Wright@mssm.edu (R.O.W.); Chris.Gennings@mssm.edu (C.G.); 2 Instituto Nacional de Salud Pública, Avenida Universidad 655, Santa María Ahuacatitlán, Cuernavaca 62100, Mexico; alejandra.cantoral@insp.mx (A.J.C.); mmtellez@insp.mx (M.M.T.-R.); 3 Columbia Mailman School of Public Health, 722 W 168th Street, New York, NY 10032, USA; mao5@cumc.columbia.edu (M.A.O.); ab4303@cumc.columbia.edu (A.A.B.); 4 Instituto Nacional de Perinatología, Montes Urales 800, Lomas Virreyes, Ciudad de México C.P. 11000, Mexico; ivandpantic@gmail.com (I.P.); lschnaas@hotmail.com (L.S.); 5 Division of Chronic Disease Research across the Life course, Department of Population Medicine, Harvard Medical School, 25 Shattuck Street, Boston, MA 02115, USA; Emily_Oken@harvardpilgrim.org; 6 Harvard Pilgrim Health Care Institute, Landmark Center, 401 Park Dr #401, Boston, MA 02215, USA

**Keywords:** nutrition, neurodevelopment, micronutrients, macronutrients, nutrient mixtures

## Abstract

Adequate nutrition is important for neurodevelopment. Although nutrients are ingested in combination, the impact of specific nutrients within the context of a nutrient mixture has not been studied with respect to health, such as neurodevelopment. Therefore, we examined the impact of prenatal and childhood nutrient mixtures on neurodevelopmental outcomes. Participants included mother–child pairs in the Programming Research in Obesity, Growth, Environment, and Social Stress (PROGRESS) prospective birth cohort in Mexico City. We assessed prenatal and child micro- and macronutrient profiles among 65 and 329 children, respectively, via food frequency questionnaires. Neurodevelopmental outcomes of 4–6 year-old children were measured using the McCarthy Scales of Children’s Abilities (MSCA). We conducted weighted quantile sum (WQS) regression analyses to calculate indices reflecting “good” and “poor” prenatal and childhood nutrition. After adjusting for maternal education, socioeconomic status, the Home Observation for Measurement of the Environment (HOME) score, and total caloric intake, the good prenatal and childhood nutrition indices predicted more favorable neurodevelopment, while both poor nutrition indices predicted poorer neurodevelopment. These associations were stronger in prenatal than childhood models. Monounsaturated fats predicted various neurodevelopmental abilities relatively strongly in both models. Prenatal and childhood consumption of combinations of beneficial nutrients may contribute to more favorable neurodevelopment.

## 1. Introduction

A large body of research supports links between nutrition and neurodevelopment [1,2,3,4,5,6]. Adequate concentrations of vitamins, minerals, essential elements, protein, and fatty acids have been shown to have beneficial effects on the developing brain, while deficiencies of these nutrients can be harmful [7,8]. Although humans generally ingest nutrients in combination, the majority of studies on nutrition and neurodevelopment have either examined single nutrients or nutrient ratios [1,3,4,9]. This approach assumes that each nutrient acts independently, but there may be interactions that explain much more of the variance. Finally, the current paradigm does not reflect the real-life scenario of food consumption, in which nutrients are always consumed as mixtures. Therefore, we propose that research on the joint effect of nutrient mixtures on health is needed. In this study, we focus on neurodevelopmental health and early-life nutrition.

A large body of literature has linked multiple food components to health. For example, various vitamins and minerals have independently been demonstrated to play important roles in brain development [1,2,3,4,5,6]. Deficiencies in vitamins A and D have been implicated in learning and memory development, as well as language and psychomotor development, respectively [4,9]. Deficiencies in B vitamins, particularly B9 (folate), B1 (thiamine), and B12 (cobalamin), have been associated with neural tube defects, delayed language development, and impaired motor functioning, respectively [2,3]. Additionally, adequate levels of nutrient minerals such as iron, manganese, selenium, and zinc have been shown to be neuroprotective. Nonetheless, while we require sufficient levels of many nutrients, nutrients can be toxic when consumed in excess. Thus, both deficiency and excess consumption can adversely affect neurodevelopment [4,5,6,10]. 

Protein and polyunsaturated fatty acids are essential macronutrients for brain growth and neuron differentiation. Protein is fundamental for neurotransmitter production as well as synapse and dendrite formation [7], and polyunsaturated fats contribute to neural cellular membrane volume and fluidity, protein enzyme activation, and neuron signal transduction [11]. Expectedly, adequate consumption of both macronutrients during early development has been shown to contribute to more favorable neurodevelopmental outcomes among preterm or low-birth-weight infants [12,13]. 

Some studies have examined nutrition profiles among children with neurodevelopmental disorders. They show that poor nutrition intake (according to the recommended dietary allowance and estimated average requirement guidelines) and multiple, rather than single, nutrient deficiencies are more common among these children and, in some cases, their mothers [14,15]. While not in and of itself proof of interactions among nutrient mixtures, this finding may be consistent with a nutrient mixture’s effect on neurodevelopment. In addition, while these studies did not examine prenatal nutrition profiles, the findings could potentially point to a role of general poor nutrition in the etiology and/or course of neurodevelopmental disorders. Prospective studies are needed to address this issue, as developmental disorders themselves may alter diet, through altered child behavior, parental nonmedical interventions, pica behaviors, or changes in appetite.

Therefore, we examined the impact of prenatal and childhood nutrient mixtures on child neurodevelopment. We hypothesized that prenatal and childhood diets rich in beneficial nutrients would positively predict child neurodevelopmental outcomes, while diets lower in these nutrients and/or higher in sugar, sodium, and/or saturated fat would negatively predict child neurodevelopmental outcomes.

## 2. Materials and Methods

### 2.1. Participants

The Programming Research in Obesity, Growth, Environment, and Social Stress (PROGRESS) is a prospective birth cohort based in Mexico City, consisting of 760 actively followed mother–child pairs. Detailed participant recruitment and follow-up procedures are reported elsewhere [16]. Briefly, women who received health insurance and prenatal care through the Mexican Social Security System (IMSS) were recruited between 2007 and 2011, and provided their written informed consent. Inclusion criteria were being at less than 20 weeks gestation at the start of the study, being at least 18 years of age, and planning to reside in Mexico City for the next three years. Women who had heart or kidney disease, used steroids or anti-epilepsy drugs, or consumed alcohol on a daily basis were not eligible.

Participants were followed during pregnancy and their children were enrolled at birth and followed longitudinally thereafter. There were 948 mothers who gave birth to a live infant and were initial cohort members; however, 760 are still actively followed. Approximately 600–650 attended timed study visits at 2, 4, and 6 years of age. Data were collected via questionnaires, biological samples, in-person measurements, and psychometric testing that was administered in a standardized manner by psychologists. The PROGRESS study was conducted in accordance with the Declaration of Helsinki, and institutional Review Board approval was granted by The Icahn School of Medicine at Mount Sinai, Harvard School of Public Health, The National Institute of Public Health in Mexico, and the Brigham and Women’s Hospital.

### 2.2. Nutrition Measures

A one-month-recall food frequency questionnaire (FFQ) was administered to a subset of 120 mothers during their third trimester. The FFQ was validated for the Mexican population [17] and administered by trained personnel in a standardized manner. It included 116 foods that were most representative of local consumption grouped into 10 categories (dairy products, fruits, vegetables, legumes, cereals, sweets, beverages, fats, snacks, and eggs and meats). The questionnaire included 10 frequency values ranging from never consumed to consumed 6 or more times daily. Nutritional vectors were obtained from food composition tables supplied by the U.S. Department of Agriculture (USDA) [18] and the Mexican National Institute of Nutrition and Medical Sciences Salvador Zubirán (INNCMNSZ) [19,20]. Additionally, an FFQ was administered to mothers assessing their child’s dietary patterns at ages 4 and 6. The child FFQ involves 1-week recall and includes 101 foods grouped into 14 categories [20]. Both FFQs also asked about supplement use. Ninety-five percent of pregnant women reported using supplements, while only 5% of children were reported to have been using supplements.

After completion of the FFQ, a nutrient analysis software program created by our research group was used to obtain nutrition vectors [20]. Since information was organized by week of intake, total quantities of energy and nutrients were divided by 7 to obtain average daily intakes. Prenatal nutrient intakes were measured from food and supplement sources since almost all women reported supplement use. Conversely, child nutrient intakes were measured exclusively from food since few children were reported to have been using supplements.

### 2.3. Neurodevelopmental Outcome Measures

Children’s cognitive abilities were tested using the McCarthy Scales of Children’s Abilities (MSCA) [21]. Cognitive testing and the childhood FFQ were administered on the same day; however, the FFQ measured eating habits from the prior week. The MSCA is a valid and reliable measure of verbal, perceptual, quantitative, motor, and memory abilities. The MSCA version used had been translated from English to Spanish and normalized for Spanish-speaking children 2.5 to 8.5 years old [22]. It was administered by psychologists at the National Institute of Perinatology. The MSCA consists of 6 scales, including: Verbal (assesses the child’s ability to vocally express thoughts and understand verbal stimuli), Perceptual Performance (assesses visual–motor coordination and nonverbal reasoning), Quantitative (assesses understanding of quantitative concepts and facility in working with numbers), Memory (assesses short-term memory of auditory and visual stimuli), Motor (assesses fine and gross motor coordination), and the General Cognitive Index (GCI; a composite of verbal, perceptual performance, and quantitative scales) [23].

### 2.4. Covariates

Covariates empirically demonstrated in the scientific literature to relate to neurodevelopment were selected a priori. Included in the final model were maternal education, socioeconomic status, the Home Observation for Measurement of the Environment (HOME) score at 24 months postpartum, and total maternal or child caloric intake, depending on the analysis.

Maternal education was categorized into: less than secondary school, secondary school, or greater than secondary school. For socioeconomic status, an algorithm was used to calculate a six-level index variable that included: education of the head of household; number of rooms, bathrooms, and showers in the house; and whether the household had an automatic washing machine, videocassette recorder, toaster, vacuum cleaner, microwave oven, and personal computer. The index was then collapsed into three categories representing low, middle, and high socioeconomic status [24]. The HOME inventory assesses the quality and quantity of stimulation and support that a child has access to within the home environment [25]. Home scores were measured in the participants’ home during a separate visit. 

### 2.5. Statistical Methods

#### 2.5.1. Nutrition Indices

To account for the complex correlation structure among nutrient mixtures, we applied a weighted quantile sum (WQS) regression [26] approach to estimate and examine “good” and “poor” nutrition indices. In creating our indices, we assumed a monotonic relationship between nutrients and neurodevelopment. A range of macro- and micronutrients were selected a priori, and all selected nutrients were ranked into deciles. Higher or lower levels of specific nutrients were classified as “good” or “poor” based on U.S. dietary guidelines [27]. We utilized U.S. dietary guidelines because they are organized according to specific nutrients, whereas Mexican guidelines are organized according to food groups. 

Nutrients demonstrated previously to have beneficial effects on prenatal brain development and/or overall health were included in the good nutrition index [7,8]. These included: vitamins, minerals, unsaturated fats, and protein (see Appendix A). In the good nutrition index, relatively higher levels of these beneficial nutrients received higher ranks. Conversely, in the poor nutrition index, higher levels of nutrients considered less health-promoting, such as sugar, sodium, and saturated fat, as well as the negated values of beneficial nutrients, received higher ranks (see Appendix A). Therefore, relative to the rest of the sample, diets richer in vitamins, minerals, protein, and unsaturated fats received a higher composite score on the good nutrition index, while relative to the rest of the sample, diets lower in beneficial nutrients and higher in saturated fat, sodium, and/or sugar received a higher composite score in the poor nutrition index. 

#### 2.5.2. Analytic Samples

The first analytic sample examined the association between maternal nutrition and child MSCA scores at 4 to 6 years of age. It consisted of the 120 mothers who were administered the FFQ during the third trimester of pregnancy. Of these 120 mothers, 66 had a child who received MSCA scores and had complete covariate data. One mother-child pair was excluded because maternal daily caloric intake was an extreme outlier (5100 calories) and possibly a fieldwork error. Therefore, the final longitudinal sample consisted of 65 mother-child pairs. 

The second analytic sample examined the cross-sectional association between children’s nutrition and their MSCA scores at 4 to 6 years of age. In total, 591 children, including the 65 children who were in the first analytic sample, had FFQ and MSCA data. The main analysis was conducted on a subset of 329 children who had all measured variables as well as FFQ energy values within 50% of their target daily calories based on body size and age [28]. We conducted a sensitivity analysis in which we imputed missing data for HOME score since this covariate contributed the most to our reduction in sample size, but results did not significantly change when data was imputed.2.5.3. Statistical Analyses.

Prior to data analysis, we attempted to split the data into a training set to estimate the weights per component and a validation set for testing the significance of the regression coefficient associated with the index; however, the weights were unstable due to small sample sizes for both analytic samples. Therefore, we did not split the data. 

WQS regression analyses resulted in an empirically weighted sum of quantiles of the food components, estimated across 100 bootstrap samples for each outcome subtest scale. A regression model was created using this empirically determined WQS index as well as covariates. When the WQS index was significant, corresponding weights were examined to identify the relative contribution of each nutrient within the index to the outcome. The WQS regression method is robust to the improper assignment of a nutrient as good or poor because that nutrient would be assigned a negligible weight within the WQS regression model, minimizing potential error in nutrient grouping. An alpha of 0.05 was the criterion for statistical significance. All statistical analyses were conducted with SAS statistical analysis software version 9.4 (SAS, Cary, NC, USA).

## 3. Results

Demographic characteristics and means and standard deviations of MSCA scores for prenatal and childhood samples are included in Table 1. Descriptive statistics for prenatal and childhood micronutrient and macronutrient intakes, along with the U.S. dietary reference ranges for each nutrient [18], are presented in Table 2. Most mean nutrient intakes tended to be adequate as per the recommended reference range; however, each nutrient had participants (9–98.5% of the population) with levels below this recommended range. Conversely, most nutrients did not have participants with levels above the upper tolerable limit. For nutrients that did, these individuals comprised no more than 5% of the study population, with the exception of iron, for which 74% of women exceeded the upper tolerable intake limit as a result of supplement use. 

### 3.1. Prenatal Nutrition

**Figure 1:**
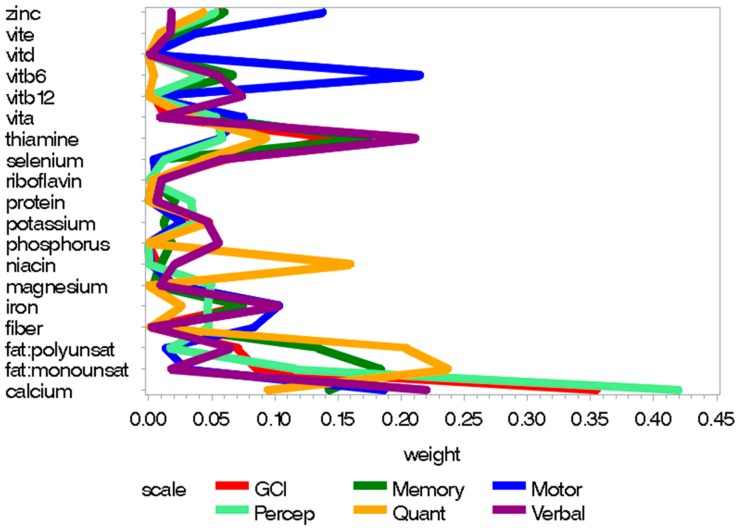
Weight plots for statistically significant scales from the prenatal “good nutrition” index, including dietary and supplemental nutrition intake.

The good prenatal nutrition index significantly positively predicted scores on all MSCA scales (see Table 3). The WQS regression weight plots, reflecting the relative contribution of each nutrient within the mixture to MSCA scores, are presented in Figure 1. Thiamine had a relatively high weight within the mixture when predicting verbal scores, and monounsaturated fatty acids had high weights relative to other nutrients when predicting memory and quantitative scores. Vitamin B6 also had a higher weight when predicting motor development, and other nutrients had non-negligible weights. Additionally, calcium had a relatively high weight when predicting all scales; particularly perceptual performance and the GCI. 

**Figure 2:**
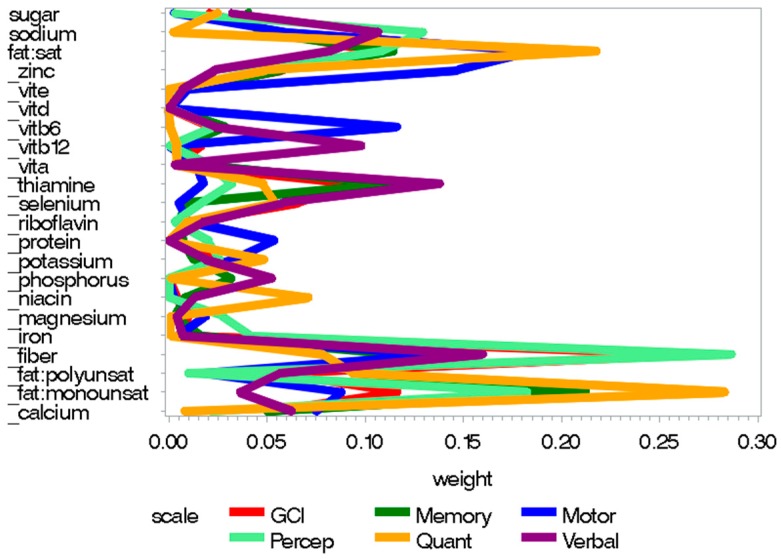
Weight plots for statistically significant scales from the prenatal “poor nutrition” index, including dietary and supplemental nutrition intake.

The poor prenatal nutrition index was significantly negatively associated with all of the MSCA scales (Table 3). Lower fiber intake had a relatively high weight when predicting poorer perceptual performance as well as poorer GCI scores. Additionally, higher saturated fat consumption and lower monounsaturated fat consumption had higher weights when predicting poorer quantitative and motor scores, and poorer quantitative and memory scores, respectively. Lower thiamine intake also had higher weights when predicting poorer verbal scores (see Figure 2). Weights per nutrient for statistically significant scales from prenatal good and poor nutrition indices are presented in Supplementary Tables S1 and S2 respectively See Supplementary Figure S1 for individual-level data from prenatal nutrition analyses. Figure legends are presented in Appendix B.

### 3.2. Childhood Nutrition

**Figure 3:**
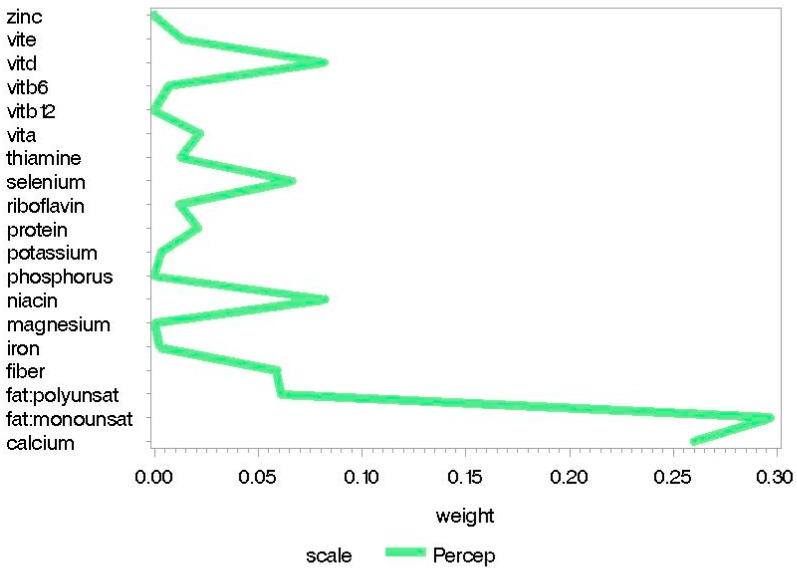
Weight plots for statistically significant scales from the childhood “good nutrition” index of dietary nutrition intake.

**Figure 4:**
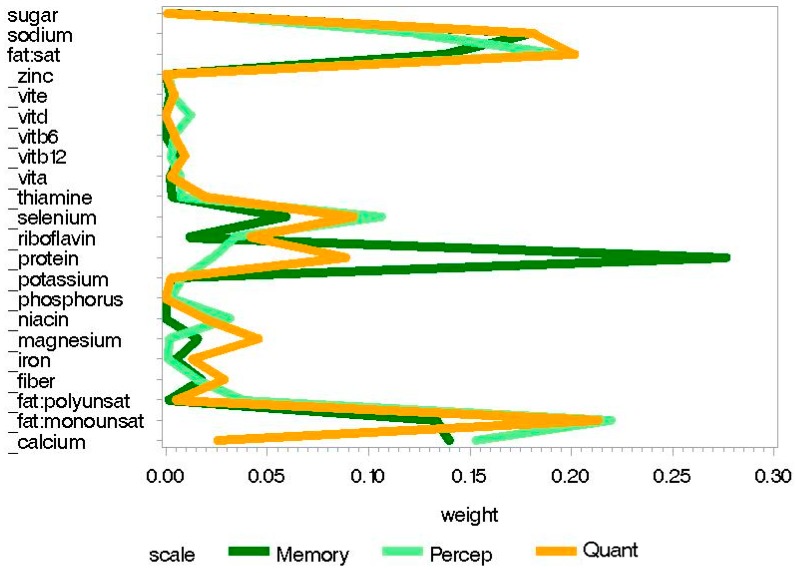
Weight plots for statistically significant scales from the childhood “poor nutrition” index of dietary nutrition intake.

The childhood good nutrition index was significantly positively associated with perceptual performance scores, while the childhood poor nutrition index was significantly negatively associated with memory, quantitative, and perceptual performance scores (see Table 4). Associations between nutrition indices and neurodevelopment were weaker for the childhood models than for the prenatal models. In the good nutrition index, monounsaturated fat and calcium had relatively high weights when predicting more favorable perceptual performance. In the poor nutrition index, higher sodium and saturated fat consumption and lower monounsaturated fat consumption had relatively high weights when predicting poorer memory, quantitative, and perceptual performance scores. Lower protein intake also had a higher weight when predicting poorer memory scores (see Figure 3 and Figure 4). Weights per nutrient for statistically significant scales from childhood good and poor nutrition indices are presented in Supplementary Tables S3 and S4 respectively. See Supplementary Figure S2 for individual-level data from childhood nutrition analyses. Figure legends are presented in Appendix B.

## 4. Discussion

All diets are a mixture of nutrients in various relative doses. This complex covariance structure has long been an unmeasured source of variance in the study of health effects and individual nutrients. To our knowledge, this is the first study to examine the relationship between maternal and child nutrient mixtures and a well-known health effect sensitive to nutrition: child neurodevelopment. We estimated the overall mixture effect of nutrients and the relative contribution of each nutrient to the children’s McCarthy Scales of Children’s Abilities (MSCA) scores, using data from empirically validated food frequency questionnaires. Results for both good and poor nutrition indices support the validity of this approach as we found significant associations in the expected directions.

As hypothesized, mothers who consumed more nutritious diets during pregnancy tended to have children with more favorable neurodevelopmental outcomes, while mothers who consumed less nutritious diets and/or higher levels of sodium, saturated fat, and/or sugar during pregnancy tended to have children with poorer neurodevelopmental outcomes. This suggests that the consumption of more comprehensively nutritious prenatal diets favorably affects child neurodevelopment, while the consumption of less comprehensively nutritious prenatal diets may hinder it. Our finding that certain prenatal micronutrients contributed more to relationships with specific skills and abilities than others shows consistency with prior literature. For example, research has linked infant thiamine intake with verbal development [3]. Additionally, vitamin B6, which contributed strongly to the prenatal mixture association with motor abilities, has previously been implicated in infant gross motor development [29]. Conversely, certain macronutrients may have more global neurodevelopmental effects. The finding that monounsaturated fats were important for the mixture association with memory, quantitative, and perceptual development is consistent with their role in comprising the structural and functional foundation of the developing brain [30]. Although fiber, which contributed strongly to the mixture association for various neurodevelopmental abilities, has not been directly examined in this regard, it positively contributes to overall microbiome function [31], and thus may indirectly facilitate neurodevelopment by enhancing the absorption of other nutrients that are important for it or by altering the microbiome to a more favorable developmental profile. 

Higher prenatal calcium also strongly predicted more favorable neurodevelopment, and there are several mechanisms by which it may contribute. First, calcium supplements have been shown to reduce blood lead among pregnant women in Mexico [32], which may indirectly improve the neurodevelopment of offspring. Second, calcium may positively impact fetal brain development directly since it facilitates the formation of neural synaptic connections and neurotransmission [33,34]. Third, adequate calcium is important for thyroid function [35], and normal maternal thyroid function during pregnancy is important for healthy infant neurodevelopment [36]. Thus, maternal calcium could directly and indirectly contribute to infant neurodevelopment via these mechanisms.

Findings from the poor prenatal nutrition index were consistent with the good prenatal nutrition index. Lower monounsaturated fat contributed strongly to the association of the mixture with poorer quantitative and memory abilities, and lower thiamine intake contributed strongly to the association of the mixture with poorer verbal abilities. This provides further support for the importance of these nutrients for these abilities. In addition, higher saturated fat consumption within the context of the mixture predicted poorer neurodevelopment, particularly in the quantitative realm. Reasons for this association are not entirely clear; however, it could be that higher saturated fat intake contributes to maternal health problems that negatively affect fetal brain development. Still, there is evidence that saturated fats may in fact be essential to neurodevelopment or a proxy for other nutrients, such as cholesterol, that are critical to it. The APOE4 gene variant that is linked to neurodegeneration, for example, is predictive of better performance in cognitive skills in children [37] as well as higher serum levels of cholesterol and saturated fats [38]. Some research also suggests that medium-chain triglycerides found in certain foods high in saturated fat, particularly coconut oil, may have brain health benefits [39]. Therefore, more research is needed to better understand this finding. Lower fiber intake also appeared to contribute relatively strongly within the mixture to associations with various poorer neurodevelopmental outcomes. Although increased prevalence of fiber deficiency (along with other nutrient deficiencies) has been reported among children with neurodevelopmental disorders [15], future studies would be needed to determine whether this, or even lower intake within the recommended intake range, could be a contributing factor. 

Consistent with our hypothesis, children with more nutritious diets tended to have more favorable perceptual performance, while children with less nutritious diets and/or higher levels of sodium, saturated fat, and/or sugar consumption tended to perform more poorly on memory, perceptual, and quantitative tasks. These associations were weaker than in prenatal models, which could suggest that prenatal nutrition plays a greater role in neurodevelopment than childhood nutrition; however, future studies are needed to further explore this finding. Consistent with prenatal analyses, within the nutrient mixture, higher calcium and monounsaturated fat intakes appeared to be particularly important for favorable neurodevelopment, especially perception, while lower protein intake, essential for brain growth and neurotransmitter synthesis [7], contributed relatively strongly to the association with poorer memory. Higher sodium and saturated fat intake also contributed relatively strongly within the mixture to associations with poorer performance in various domains. It is possible that consuming snack foods high in sodium and saturated fat that also contain other additives and preservatives known to interfere with attentional processes [40] may have accounted for this association, or that consuming such foods is a marker of other poor health behaviors that can impact neurodevelopment. 

Our study is limited by several factors. First, given that the FFQs measured eating habits over the course of one month for women and one week for children, they may not have captured typical eating habits for people whose eating habits were uncharacteristic during that time. In addition, there may have been changes in nutrition patterns between birth and age of 4 years that could have affected neurodevelopment, but were not captured by the childhood FFQ. Questionnaires have recall error that can affect results, although we believe that this is likely to be nondifferential with respect to neurodevelopment measured at 4–5 years following the prenatal FFQ. In addition, dietary intake does not perfectly correlate with the absorption of nutrients, which is another source of nondifferential error. For example, most women consumed excess levels of iron; however, in Mexico, iron deficiency is still prevalent because the bioavailability of supplemental iron is low and phytic acid consumption is high. We did not include dietary folate intake in our models because folate concentrations from food were not distinguished from folic acid concentrations from supplements at the time of study. Lastly, the prenatal sample size was small, which may limit the generalizability of these results. Future studies examining the consumption of combinations of nutrients and neurodevelopment with larger sample sizes are needed to provide further validation for our findings. 

## 5. Conclusions

By examining nutrients as components of a larger mixture, we characterized real-life food consumption effects on neurodevelopment. We found that more nutritious prenatal or childhood diets predicted more favorable neurodevelopment. We also validated links between specific nutrients and specific neurodevelopmental abilities found in previous literature, and provided new insights into the important role of certain nutrients (within the context of a comprehensively nutritious diet) for brain development. These findings illustrate new methods that may lead to an improved understanding of the impact of nutrition profiles early in life on neurodevelopmental outcomes. 

## Figures and Tables

**Table 1:** Sociodemographic and psychometric characteristics of mothers and their children.

	Third Trimester Nutrition Sample (*n* = 65)	4–6 Year-Old Cross-Sectional Sample (*n* = 329)	Entire Cohort (*n* = 948) ^1,2^
Mother’s age at enrollment (years), mean (SD)	28.42 (6.06)	27.77 (5.57)	27.66 (5.48)
Maternal Education, n (%)			
<Secondary school	25 (39%)	129 (39%)	385 (41%)
Secondary school	21 (32%)	118 (36%)	334 (35%)
>Secondary school	19 (29%)	82 (25%)	229 (24%)
SES index, n (%)			
Low	28 (43%)	172 (52%)	486 (51%)
Medium	31 (48%)	125 (38%)	356 (38%)
High	6 (9%)	32 (10%)	106 (11%)
Sex of child, n (%)			
Male	33 (51%)	186 (57%)	498 (53%)
Female	32 (49%)	143 (43%)	450 (47%)
Child’s birthweight (kg), mean (SD)	3.10 (0.40)	3.12 (0.43)	3.04 (0.49)
Gestational age at birth (weeks), mean (SD)	38.28 (1.49)	38.49 (1.62)	38.24 (1.96)
HOME Score at 24 months, mean (SD)	33.45 (4.72)	31.91 (5.41)	31.72 (5.42)
McCarthy Scales, mean (SD)			
Memory	46.20 (7.23)	47.01 (8.15)	47.06 (8.35)
Quantitative	45.66 (7.98)	46.10 (9.19)	45.92 (9.44)
Motor	44.42 (9.85)	44.47 (8.67)	44.44 (8.61)
Perception	52.83 (7.71)	51.96 (7.57)	51.71 (7.94)
Verbal	48.91 (8.59)	49.87 (8.97)	49.81 (8.97)
GCI	99.20 (12.52)	99.78 (12.86)	99.55 (13.19)

^1^ The mean HOME score for the entire cohort is based on a sample size of 497. ^2^ The mean McCarthy Scales scores for the entire cohort are based on a sample size of 607. Abbreviations: SD, standard deviation; SES, socioeconomic status; HOME score, Home Observation for Measurement of the Environment; GCI, Global Composite Index.

**Table 2:** Prenatal and childhood dietary intake with reference to U.S. dietary guidelines.

Nutrition Component	Prenatal Nutrition (*n* = 65)					4–6 Year-Old Nutrition (*n* = 329)		
	Dietary ^1^ Intake Reference for Pregnancy	Tolerable Upper Level	Food and Supplement Intake		Child ^2^ Dietary Intake Reference^1^	Tolerable Upper Level	Food Intake ^3^	
			Mean (SD)	Range			Mean (SD)	Range
Kilocalories			2295.6 (852.2)	872.5–4839.6			1447.3 (356.2)	642.9–2417.06
Protein, g	71		74.6 (26.1)	30.2–153.4	19		47.6 (12.5)	20.0–82.5
Zinc, mg	11	40	13.9 (10.0)	3.5–43.4	5	12	6.3 (1.9)	1.8–15.4
Vitamin A, mcg RAE	770	3000	1016.8 (923.5)	332.9–7009.4	400	900	492.8 (227.9)	73.3–1383.5
Vitamin E, mg	15	1000	11.2 (4.3)	3.8–22.4	7	300	5.8 (2.6)	0.8–15.6
Vitamin D, IU	600		107.8 (159.4)	1.8–1019.4	600		43.1 (51.4)	1.3–389.2
Vitamin B6, mg	1.9	100	2.0 (0.8)	0.6–4.7	0.6	40	1.1 (0.4)	0.3–3.7
Vitamin B12, mcg	2.6		4.4 (3.4)	0.8–27.9	1.2 mcg		2.4 (1.3)	0.5–11.9
Thiamine, mg	1.4		14.2 (33.0)	0.5–103.9	0.6		0.8 (0.2)	0.2–1.9
Selenium, mcg	60	400	45.6 (25.5)	11.4–149.3	30	150	23.1 (11.0)	4.8–86.3
Riboflavin, mg	1.4		2.5 (1.7)	0.6–7.6	0.6		1.0 (0.4)	0.2–2.7
Potassium, mg	4700		3189.8 (1419.2)	852.3–9421.4	3800		1580.1 (621.9)	398.1–4369.2
Phosphorus, mg	700	3500	1334.0 (485.6)	440.5–2363.7	500	3000	893.8 (313.6)	219.5–1916.7
Niacin, mg	18	35	15.2 (5.5)	6.0–29.6	8	15	9.3 (3.2)	2.4–21.7
Magnesium, mg	350–360	350	330.0 (137.1)	85.4–778.9	130	110	178.3 (57.4)	51.5–394.9
Iron, mg	27	45	54.1 (28.4)	6.8–153.2	10	40	8.4 (3.0)	2.8–20.2
Fiber, g	28		22.0 (9.7)	5.1–54.3	16.8–19.6		11.3 (5.1)	3.0–45.1
Calcium, mg	1000	2500	1460.8 (559.1)	387.7–3064.2	1000	2500	937.1 (353.0)	170.0–2237.0
Monounsaturated fat, g			21.1 (9.8)	6.2–55.4			13.6 (5.2)	2.5–36.5
Polyunsaturated fat, g	15.5		11.3 (5.2)	2.5–24.2	10		6.7 (3.1)	1.4–28.6
Saturated fat, mg			28.6 (13.2)	9.6–64.9			17.9 (9.0)	1.9–54.0
Sugar, g			38.5 (44.9)	4.7–237.5			31.7 (23.8)	1.0–179.9
Sodium, mg	1500	2300	2241.9 (844.4)	861.4–4820.2	1200	1900	1605.4 (546.7)	581.4–3733.6
Carbohydrates, g	175		337.7 (165.6)	97.3–1008.7	130		198.9 (57.4)	81.6–421.2

^1^ Empty cell indicates no true dietary reference. References were obtained from the National Institutes of Health, Office of Dietary Supplements. ^2^ Dietary reference intake for males and females between 4–8 years of age. ^3^ Supplements were not included for 4–6 year-old nutrition because only two children used supplements. Abbreviations: RAE, Retinol Activity Equivalent; IU, International Unit; SD, standard deviation.

**Table 3:** Weighted quantile sums (WQS) regression for prenatal “good” and “poor” nutrition indices in relation to child McCarthy Scales scores.

McCarthy Scale	Good Nutrition Index			Poor Nutrition Index		
	WQS Beta	95% CI	*p* Value	WQS Beta	95% CI	*p* Value
Memory	2.56	0.99, 4.13	0.001	−3.55	−5.71, −1.38	0.001
Quantitative	1.78	0.11, 3.44	0.037	−3.39	−6.02, −0.76	0.012
Motor	2.84	1.11, 4.57	0.001	−4.36	−6.76, −1.96	<0.001
Perception	1.64	0.33, 2.96	0.015	−2.75	−4.77, −0.72	0.008
Verbal	2.75	1.44, 4.06	<0.001	−3.71	−5.46, −1.96	<0.001
GCI	3.54	1.62, 5.45	<0.001	−4.69	−7.28, −2.10	<0.001

Note: Analyses are adjusted for sex, SES, maternal education, HOME score at 24 months, and total caloric intake; GCI, Global Composite Index.

**Table 4:** WQS regression for childhood “good” and “poor” nutrition indices in relation to child McCarthy Scales scores.

McCarthy Scale	Good Nutrition Index			Poor Nutrition Index		
	WQS Beta	95% CI	*p* Value	WQS Beta	95% CI	*p* Value
Memory	0.66	−0.03, 1.35	0.059	−1.44	−2.59, −0.30	0.014
Quantitative	0.44	−0.45, 1.32	0.335	−1.74	−3.27, −0.20	0.026
Motor	0.70	−0.18, 1.57	0.120	−1.18	−2.49, 0.13	0.078
Perception	0.81	0.01, 1.61	0.048	−1.88	−3.17, −0.58	0.004
Verbal	0.66	−0.13, 1.45	0.102	−0.73	−1.64, 0.18	0.115
GCI	0.92	−0.27, 2.11	0.131	−1.77	−3.66, 0.11	0.066

Note: Analyses are adjusted for sex, SES, maternal education, HOME score at 24 months, and total caloric intake; GCI, Global Composite Index.

## Supplementary Materials

The following are available online at http://www.mdpi.com/2072-6643/10/8/1093/s1, Table S1: Weights for statistically significant scales from the prenatal good nutrition index, Table S2: Weights for statistically significant scales from the prenatal poor nutrition index, Table S3: Weights for statistically significant scales from the childhood good nutrition index, Table S4: Weights for statistically significant scales from the childhood poor nutrition index, Figure S1: Individual level data for the verbal score for the prenatal (a) “good nutrition” index, and (b) “poor nutrition” index, Figure S2: Individual level data for the perception score for the childhood (a) “good nutrition” index, and (b) “poor nutrition” index.

## Appendix A

Nutrients included in the good nutrition index: Zinc, Vitamin E, Vitamin D, Vitamin B6, Vitamin B12, Vitamin A, Thiamine, Selenium, Riboflavin, Protein, Potassium, Phosphorus, Niacin, Magnesium, Iron, Fiber, Polyunsaturated Fat, Monounsaturated Fat, and Calcium.

Nutrients included in the poor nutrition index: Sugar, Sodium, Saturated Fat, and negated values of: Zinc, Vitamin E, Vitamin D, Vitamin B6, Vitamin B12, Vitamin A, Thiamine, Selenium, Riboflavin, Protein, Potassium, Phosphorus, Niacin, Magnesium, Iron, Fiber, Polyunsaturated Fat, Monounsaturated Fat, and Calcium.

## Appendix B

Figure Legend

- Vite = Vitamin E
- Vitd = Vitamin D
- Vitb6 = Vitamin B6
- Vitb12 = Vitamin B12
- Vita = Vitamin A
- Fat: Polyunsat = Polyunsaturated fat
- Fat: Monounsat = Monounsaturated fat
- Fat: Sat = Saturated Fat
- GCI = Global Composite Index
- Percep = Perceptual Performance
- Quan = Quantitative
- _zinc = negated value of zinc
- _vite = negated value of vitamin E
- _vitd = negated value of vitamin D
- _vitb6 = negated value of vitamin B6
- _vitb12 = negated value of vitamin B12
- _vita = negated value of vitamin A
- _thiamine = negated value of thiamine
- _selenium = negated value of selenium
- _riboflavin = negated value of riboflavin
- _protein = negated value of protein
- _potassium = negated value of potassium
- _phosphorus = negated value of phosphorus
- _niacin = negated value of niacin
- _magnesium = negated value of magnesium
- _iron = negated value of iron
- _fiber = negated value of fiber
- _fat: Polyunsat = negated value of polyunsaturated fat
- _fat: Monounsat = negated value of monounsaturated fat
- _calcium = negated value of calcium
- Adj = Adjusted

## References

[1] Yang X., Bao Y., Fu H., Li L., Ren T., Yu X. (2014). Selenium protects neonates against neurotoxicity from prenatal exposure to manganese. PLoS ONE.

[2] Black M.M. (2008). Effects of vitamin B12 and folate deficiency on brain development in children. Food Nutr. Bull..

[3] Fattal-Valevski A., Azouri-Fattal I., Greenstein Y.J., Guindy M., Blau A., Zelnik N. (2009). Delayed language development due to infantile thiamine deficiency. Dev. Med. Child Neurol..

[4] Zhu P., Tong S.L., Hao J.H., Tao R.X., Huang K., Hu W.B., Zhou Q.F., Jiang X.M., Tao F.B. (2015). Cord blood vitamin D and neurocognitive development are nonlinearly related in toddlers. J. Nutr..

[5] Christian P., Murray-Kolb L.E., Khatry S.K., Katz J., Schaefer B.A., Cole P.M., Leclerq S.C., Tielsch J.M. (2010). Prenatal micronutrient supplementation and intellectual and motor function in early school-aged children in nepal. JAMA.

[6] Yang X., Yu X., Fu H., Li L., Ren T. (2013). Different levels of prenatal zinc and selenium had different effects on neonatal neurobehavioral development. Neurotoxicology.

[7] Georgieff M.K. (2007). Nutrition and the developing brain: Nutrient priorities and measurement. Am. J. Clin. Nutr..

[8] Gonzalez H.F., Visentin S. (2016). Micronutrients and neurodevelopment: An update. Arch. Argent. Pediatr..

[9] Hou N., Ren L., Gong M., Bi Y., Gu Y., Dong Z., Liu Y., Chen J., Li T. (2015). Vitamin A deficiency impairs spatial learning and memory: The mechanism of abnormal CBP-dependent histone acetylation regulated by retinoic acid receptor alpha. Mol. Neurobiol..

[10] Chung S.E., Cheong H.K., Ha E.H., Kim B.N., Ha M., Kim Y., Hong Y.C., Park H., Oh S.Y. (2015). Maternal blood manganese and early neurodevelopment: The mothers and children’s environmental health (MOCEH) study. Environ. Health Perspect..

[11] Uauy R., Dangour A.D. (2006). Nutrition in brain development and aging: Role of essential fatty acids. Nutr. Rev..

[12] Clandinin M.T., Van Aerde J.E., Merkel K.L., Harris C.L., Springer M.A., Hansen J.W., Diersen-Schade D.A. (2005). Growth and development of preterm infants fed infant formulas containing docosahexaenoic acid and arachidonic acid. J. Pediatr..

[13] Stephens B.E., Walden R.V., Gargus R.A., Tucker R., McKinley L., Mance M., Nye J., Vohr B.R. (2009). First-week protein and energy intakes are associated with 18-month developmental outcomes in extremely low birth weight infants. Pediatrics.

[14] Keen C.L., Uriu-Adams J.Y., Skalny A., Grabeklis A., Grabeklis S., Green K., Yevtushok L., Wertelecki W.W., Chambers C.D. (2010). The plausibility of maternal nutritional status being a contributing factor to the risk for fetal alcohol spectrum disorders: The potential influence of zinc status as an example. Biofactors.

[15] Nguyen T.T., Risbud R.D., Chambers C.D., Thomas J.D. (2016). Dietary nutrient intake in school-aged children with heavy prenatal alcohol exposure. Alcohol. Clin. Exp. Res..

[16] Hernandez M., Aguirre J., Serrano L. (1983). Alimentacio’n de Obreros Y Sus Familias.

[17] Hernandez-Avila M., Romieu I., Parra S., Hernandez-Avila J., Madrigal H., Willett W. (1998). Validity and reproducibility of a food frequency questionnaire to assess dietary intake of women living in Mexico city. Salud Publ. Mex..

[18] USDA Dietary Supplement Fact Sheets. https://ods.od.nih.gov/factsheets/list-all/.

[19] Bourges H. (1996). Tablas de Composicio’n de Alimentos. “50 Aniversario Instituto Nacional de la Nutricio’n”.

[20] Rodriguez-Ramirez S., Mundo-Rosas V., Jimenez-Aguilar A., Shamah-Levy T. (2009). Methodology for the analysis of dietary data from the Mexican national health and nutrition survey 2006. Salud Publ. Mex..

[21] McCarthy D. (1972). Manual for the Mccarthy Scales of Children’s Abilities.

[22] McCarthy D. Escalas Mccarthy de Aptitudes Y Psicomotricidad Para Niños. http://www.pearsonclinical.es/producto/63/msca-escalas-mccarthy-de-aptitudes-y-psicomotricidad-para-ninos.

[23] Kaufman A.S., Kaufman N.L. (1977). Clinical Evaluation of Young Children with the McCarthy Scales.

[24] Carrasco A.V. The AMAI System of Classifying Households by Socio-Economic Level: The Experience of Mexico and Its Comparison with Brazil and Argentina. Proceedings of the Latin American Conference.

[25] Caldwell B., Bradley R. (2003). Home Observation for Measurement of the Environment: Administration Manual.

[26] Carrico C., Gennings C., Wheeler D.C., Factor-Litvak P. (2015). Characterization of weighted quantile sum regression for highly correlated data in a risk analysis setting. J. Agric. Biol. Environ. Stat..

[27] USDA 2015–2020 Dietary Guidelines for Americans, 8th ed. http://health.gov/dietaryguidelines/2015/guidelines/.

[28] Ramirez-Silva I., Jimenez-Aguilar A., Valenzuela-Bravo D., Martinez-Tapia B., Rodriguez-Ramirez S., Gaona-Pineda E.B., Angulo-Estrada S., Shamah-Levy T. (2016). Methodology for estimating dietary data from the semi-quantitative food frequency questionnaire of the Mexican national health and nutrition survey 2012. Salud Publ. Mex..

[29] Torsvik I.K., Ueland P.M., Markestad T., Midttun O., Bjorke Monsen A.L. (2015). Motor development related to duration of exclusive breastfeeding, B vitamin status and B12 supplementation in infants with a birth weight between 2000–3000 g, results from a randomized intervention trial. BMC Pediatr..

[30] Martínez M., Mougan I. (1998). Fatty acid composition of human brain phospholipids during normal development. J Neurochem..

[31] Statovci D., Aguilera M., MacSharry J., Melgar S. (2017). The impact of western diet and nutrients on the microbiota and immune response at mucosal interfaces. Front. Immunol..

[32] Ettinger A.S., Lamadrid-Figueroa H., Tellez-Rojo M.M., Mercado-Garcia A., Peterson K.E., Schwartz J., Hu H., Hernandez-Avila M. (2009). Effect of calcium supplementation on blood lead levels in pregnancy: A randomized placebo-controlled trial. Environ. Health Perspect..

[33] Lohmann C. (2009). Calcium signaling and the development of specific neuronal connections. Prog. Brain Res..

[34] Neher E., Sakaba T. (2008). Multiple roles of calcium ions in the regulation of neurotransmitter release. Neuron.

[35] Kališnik M., Zorc-Pleskovič R., Pajer Z., Pavlin K. (1990). The effect of chronic hypercalcemia or hypocalcemia on the follicular and parafollicular cells in rat thyroid gland. Am. J. Anat..

[36] Klein R.Z., Sargent J.D., Larsen P.R., Waisbren S.E., Haddow J.E., Mitchell M.L. (2001). Relation of severity of maternal hypothyroidism to cognitive development of offspring. J. Med. Screen..

[37] Wright R.O., Hu H., Silverman E.K., Tsaih S.W., Schwartz J., Bellinger D., Palazuelos E., Weiss S.T., Hernandez-Avila M. (2003). Apolipoprotein E genotype predicts 24-month bayley scales infant development score. Pediatr. Res..

[38] Bennet A.M., Di Angelantonio E., Ye Z., Wensley F., Dahlin A., Ahlbom A., Keavney B., Collins R., Wiman B., de Faire U. (2007). Association of apolipoprotein E genotypes with lipid levels and coronary risk. JAMA.

[39] Fernando W.M., Martins I.J., Goozee K.G., Brennan C.S., Jayasena V., Martins R.N. (2015). The role of dietary coconut for the prevention and treatment of alzheimer’s disease: Potential mechanisms of action. Br. J. Nutr..

[40] Boris M., Mandel F.S. (1994). Foods and additives are common causes of the attention deficit hyperactive disorder in children. Ann. Allergy.

