# Supplementary material for: Quality of Prenatal and Childhood Diet Predicts Neurodevelopmental Outcomes among Children in Mexico City
Source: Nutrients. 2018 Aug 15;10(8):1093. doi: 10.3390/nu10081093 (PMC6115750; doi:10.3390/nu10081093)
Supplement: Supplementary file 1 [file nutrients-10-01093-s001.docx]

**Table S1.** Weights for statistically significant McCarthy scales from the prenatal good nutrition index.

| **Nutrient** | **GCI** | **Memory** | **Motor** | **Perception** | **Quantitative** | **Verbal** |
| --- | --- | --- | --- | --- | --- | --- |
| Zinc | 0.056 | 0.060 | 0.138 | 0.053 | 0.043 | 0.018 |
| Vitamin E | 0.013 | 0.026 | 0.037 | 0.012 | 0.009 | 0.017 |
| Vitamin D | 0.000 | 0.001 | 0.001 | 0.000 | 0.000 | 0.001 |
| Vitamin B6 | 0.041 | 0.067 | 0.215 | 0.042 | 0.004 | 0.055 |
| Vitamin B12 | 0.005 | 0.002 | 0.000 | 0.000 | 0.000 | 0.074 |
| Vitamin A | 0.014 | 0.045 | 0.075 | 0.053 | 0.033 | 0.009 |
| Thiamine | 0.148 | 0.174 | 0.056 | 0.058 | 0.093 | 0.211 |
| Selenium | 0.061 | 0.008 | 0.004 | 0.013 | 0.043 | 0.058 |
| Riboflavin | 0.005 | 0.003 | 0.006 | 0.001 | 0.003 | 0.010 |
| Protein | 0.008 | 0.021 | 0.003 | 0.034 | 0.000 | 0.006 |
| Potassium | 0.038 | 0.012 | 0.026 | 0.037 | 0.048 | 0.047 |
| Phosphorus | 0.000 | 0.018 | 0.000 | 0.000 | 0.000 | 0.055 |
| Niacin | 0.007 | 0.010 | 0.002 | 0.000 | 0.160 | 0.021 |
| Magnesium | 0.019 | 0.004 | 0.020 | 0.049 | 0.000 | 0.009 |
| Iron | 0.073 | 0.074 | 0.103 | 0.047 | 0.026 | 0.102 |
| Fiber | 0.002 | 0.015 | 0.084 | 0.048 | 0.001 | 0.002 |
| Polyunsaturated fats | 0.071 | 0.135 | 0.014 | 0.017 | 0.204 | 0.065 |
| Monounsaturated fats | 0.086 | 0.184 | 0.030 | 0.116 | 0.237 | 0.018 |
| Calcium | 0.355 | 0.143 | 0.187 | 0.420 | 0.094 | 0.220 |

*Note.* Includes dietary and supplemental nutrition intake.

**Table S2.** Weights for statistically significant McCarthy scales from the prenatal poor nutrition index.

| **Nutrient** | **GCI** | **Memory** | **Motor** | **Perception** | **Quantitative** | **Verbal** |
| --- | --- | --- | --- | --- | --- | --- |
| Sugar | 0.021 | 0.040 | 0.003 | 0.003 | 0.025 | 0.032 |
| Sodium | 0.054 | 0.046 | 0.059 | 0.129 | 0.002 | 0.106 |
| Saturated fat | 0.104 | 0.114 | 0.186 | 0.108 | 0.218 | 0.082 |
| _Zinc | 0.058 | 0.057 | 0.147 | 0.046 | 0.049 | 0.024 |
| _Vitamin E | 0.006 | 0.010 | 0.010 | 0.002 | 0.000 | 0.007 |
| _Vitamin D | 0.000 | 0.000 | 0.001 | 0.000 | 0.000 | 0.000 |
| _Vitamin B6 | 0.021 | 0.029 | 0.116 | 0.023 | 0.001 | 0.024 |
| _Vitamin B12 | 0.016 | 0.006 | 0.001 | 0.000 | 0.004 | 0.098 |
| _Vitamin A | 0.004 | 0.017 | 0.014 | 0.021 | 0.004 | 0.003 |
| _Thiamine | 0.094 | 0.113 | 0.017 | 0.032 | 0.048 | 0.138 |
| _Selenium | 0.066 | 0.010 | 0.005 | 0.017 | 0.054 | 0.058 |
| _Riboflavin | 0.011 | 0.005 | 0.010 | 0.003 | 0.009 | 0.018 |
| _Protein | 0.001 | 0.007 | 0.053 | 0.020 | 0.000 | 0.000 |
| _Potassium | 0.025 | 0.013 | 0.033 | 0.026 | 0.048 | 0.019 |
| _Phosphorus | 0.001 | 0.032 | 0.000 | 0.000 | 0.001 | 0.052 |
| _Niacin | 0.004 | 0.008 | 0.003 | 0.000 | 0.071 | 0.013 |
| _Magnesium | 0.010 | 0.003 | 0.019 | 0.028 | 0.001 | 0.004 |
| _Iron | 0.010 | 0.016 | 0.005 | 0.041 | 0.001 | 0.007 |
| _Fiber | 0.271 | 0.118 | 0.137 | 0.287 | 0.078 | 0.160 |
| _Polyunsaturated fat | 0.055 | 0.094 | 0.018 | 0.010 | 0.094 | 0.057 |
| _Monounsaturated fat | 0.116 | 0.212 | 0.088 | 0.182 | 0.283 | 0.036 |
| _Calcium | 0.052 | 0.050 | 0.075 | 0.022 | 0.008 | 0.062 |

*Note.* Includes dietary and supplemental nutrition intake; “_” in front of nutrient denotes negated value.

**Table S3.** Weights for statistically significant McCarthy scales from the childhood good nutrition index.

| **Nutrient** | **Perception** |
| --- | --- |
| Zinc | 0.000 |
| Vitamin E | 0.013 |
| Vitamin D | 0.082 |
| Vitamin B6 | 0.007 |
| Vitamin B12 | 0.000 |
| Vitamin A | 0.022 |
| Thiamine | 0.013 |
| Selenium | 0.066 |
| Riboflavin | 0.012 |
| Protein | 0.021 |
| Potassium | 0.004 |
| Phosphorus | 0.000 |
| Niacin | 0.082 |
| Magnesium | 0.001 |
| Iron | 0.003 |
| Fiber | 0.059 |
| Polyunsaturated fat | 0.061 |
| Monounsaturated fat | 0.297 |
| Calcium | 0.260 |

Note. Includes dietary nutrition intake.

**Table S4**. Weights for statistically significant McCarthy scales from the childhood poor nutrition index

| **Nutrient** | **Memory** | **Perception** | **Quantitative** |
| --- | --- | --- | --- |
| Sugar | 0.004 | 0.009 | 0.001 |
| Sodium | 0.179 | 0.124 | 0.182 |
| Saturated fat | 0.137 | 0.194 | 0.202 |
| _Zinc | 0.000 | 0.001 | 0.000 |
| _Vitamin E | 0.002 | 0.003 | 0.004 |
| _Vitamin D | 0.000 | 0.012 | 0.000 |
| _Vitamin B6 | 0.000 | 0.004 | 0.004 |
| _Vitamin B12 | 0.006 | 0.003 | 0.009 |
| _Vitamin A | 0.002 | 0.007 | 0.003 |
| _Thiamine | 0.003 | 0.007 | 0.019 |
| _Selenium | 0.059 | 0.106 | 0.092 |
| _Riboflavin | 0.012 | 0.034 | 0.042 |
| _Protein | 0.276 | 0.023 | 0.089 |
| _Potassium | 0.006 | 0.008 | 0.003 |
| _Phosphorus | 0.001 | 0.003 | 0.000 |
| _Niacin | 0.000 | 0.031 | 0.019 |
| _Magnesium | 0.016 | 0.002 | 0.046 |
| _Iron | 0.005 | 0.001 | 0.013 |
| _Fiber | 0.018 | 0.015 | 0.029 |
| _Polyunsaturated fat | 0.001 | 0.038 | 0.005 |
| _Monounsaturated fat | 0.134 | 0.220 | 0.213 |
| _Calcium | 0.140 | 0.153 | 0.026 |

*Note*. Includes dietary nutrition intake; “_” in front of nutrient denotes negated value.


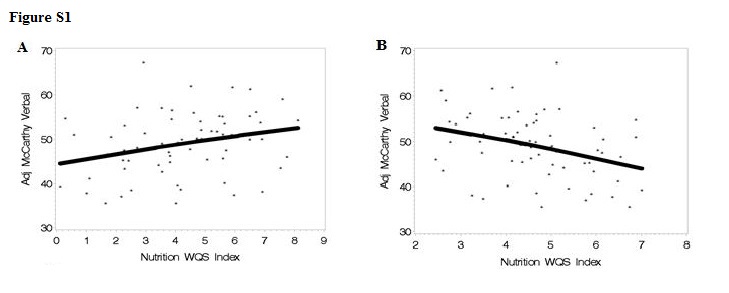


**Figure S1.** Individual level data for the verbal score for the prenatal (a) “good nutrition” index, and (b) “poor nutrition” index.


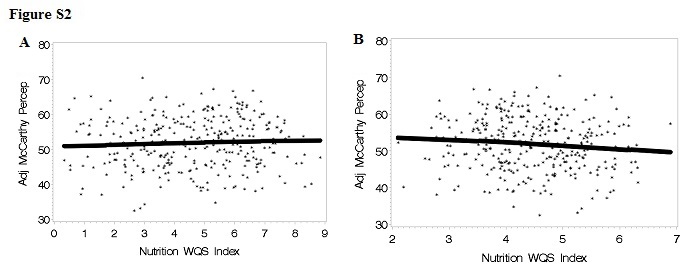


**Figure S2.** Individual level data for the perception score for the childhood (a) “good nutrition” index, and (b) “poor nutrition” index.
